# MR-compatible optical microscope for in-situ dual-mode MR-optical microscopy

**DOI:** 10.1371/journal.pone.0250903

**Published:** 2021-05-10

**Authors:** Matthias C. Wapler, Frederik Testud, Patrick Hucker, Jochen Leupold, Dominik von Elverfeldt, Maxim Zaitsev, Ulrike Wallrabe

**Affiliations:** 1 Department of Microsystemes Engineering (IMTEK), Laborarory for Microactuators, University of Freiburg, Freiburg, Germany; 2 Center for Diagnostic and Therapeutic Radiology, Medical Physics, Medical Center—University of Freiburg, Faculty of Medicine, University of Freiburg, Freiburg, Germany; 3 Center for High-Field Magnetic Resonance, Center for Medical Physics and Biomedical Engineering, Medical University of Vienna, Vienna, Austria; Nicolaus Copernicus University, POLAND

## Abstract

We present the development of a dual-mode imaging platform that combines optical microscopy with magnetic resonance microscopy. Our microscope is designed to operate inside a 9.4T small animal scanner with the option to use a 72mm bore animal RF coil or different integrated linear micro coils. With a design that minimizes the magnetic distortions near the sample, we achieved a field inhomogeneity of 19 ppb RMS. We further integrated a waveguide in the optical layout for the electromagnetic shielding of the camera, which minimizes the noise increase in the MR and optical images below practical relevance. The optical layout uses an adaptive lens for focusing, 2 × 2 modular combinations of objectives with 0.6mm to 2.3mm field of view and 4 configurable RGBW illumination channels and achieves a plano-apochromatic optical aberration correction with 0.6μm to 2.3μm resolution. We present the design, implementation and characterization of the prototype including the general optical and MR-compatible design strategies, a knife-edge optical characterization and different concurrent imaging demonstrations.

## Introduction

Two areas in magnetic resonance (MR) imaging (MRI) with great potential are MR-microscopy and multimode imaging. MR-microscopy [1] allows—with conventional spatial encoding by magnetic field gradients—to observe samples of the mm and sub-mm-scale with a resolution down to the μm-scale to image, e.g., single cells [2, 3] or fine biological structures [4–6]. While isotropic resolutions at room temperature around 3μm [7, 8] and anisotropic resolutions of 1 × 1 × 75μm^3^ [9] have been achieved, they require very small radio frequency (RF) coils of a few 100μm diameter; the resolution decreases with larger RF coils and hence larger sample volumes [10]. These resolutions are still significantly above optical microscopy and require long observation times. Multimode imaging may combine the advantages of both techniques.

After the multimodal combination of positron emission tomography (PET) and computed tomography (CT) has become common in medical imaging to combine functional and anatomic imaging, PET is also combined with MRI when MRI is the preferred anatomical imaging technique [11, 12]. The combination of MRI with optical techniques in or near the visible spectrum such as fluorescence or bioluminiscence imaging [13, 14] is, however, of limited use for conventional (animal or human) imaging because the high scattering and hence limited penetration depth [15] are a limiting factor in large objects unless we perform endoscopy or similar techniques. In these cases, the optical technique provides a lower resolution—a few mm [15]—than the MRI.

For MR microscopy, this is different: Optical microscopy can penetrate often reasonably well into the sample, it provides a resolution that is by far superior to the MR resolution and there are techniques such as fluorescence or staining to highlight particular structures. Furthermore, there is a large number of modern techniques for super-resolution and three-dimensional imaging. Hence, the combination with optical microscopy is used in most cases to identify structures and orientation of the sample in the MR image (e.g. [6]) and in some cases to explicitly correlate an optical image with the MR image [16, 17]. This is, however, usually done with an ordinary microscope ex-situ with a fixated object.

Another approach is in-situ optical observation. This has the advantage that the MR image is acquired simultaneously with the optical image, such that the object does not need to be fixated and the exact alignment of the sample is not critical. Furthermore, it can provide real-time information, for example in the case of living specimen as we further discuss in the summary and outlook section.

In the literature, there are, however, only very few examples where in-situ optical microscopy in MR settings has been attempted. In [18], the authors mounted a modified commercially available long-distance microscope axially in the bore of the MR magnet and used a 90° tilt mirror to observe the sample. This required a planar RF coil and a dedicated uniplanar gradient system which compromised gradient uniformity [19] and provided an optical resolution of ≳ 10μm, which is significantly above typical resolutions of high-quality optical microscopes. In addition, they observed noise artifacts in the MR image from the optical camera. Similarly, the authors of [20] mounted an endoscope with a tilt mirror above the sample. Their setup also uses a fixed focus, relays the image with an optical fiber bundle and provides nominally 80μm optical resolution. The only true MR-compatible microscope available in the literature to date is a confocal scanning microscope with a resolution of 1.2μm with a modified commercial long-distance (6mm) microscope objective (numerical aperture NA = 0.45, 2mm FoV) that is placed along the axis of the MR magnet [21, 22]. To be able to observe the sample, the authors used alternatively either a cylindrical RF-coil with a wide spacing between the coil windings wound around a glass capillary or a butterfly coil in the transverse plane. This approach allows for convenient observation along the axis of the magnet, and enabled MR-compatibility by relaying the image with a 1:1 telescope and placing the active components outside of the magnet. The drawbacks sre a lmited MR sensitivity of the coils, possible optical off-axis artefacts from the telescope and for the cylindrical coil also a reduction in the optical quality on-axis to 2 to 4.8m at different depths. A similar approach was taken in [23], where the authors perform two-photon fluorescence scanning microscopy. In contrast to the above, this method requires fluorescent markers in the object. Again, they moved all active components (laser, scanning mirrors, detector) outside of the magnet and used relay lenses, but they now observed orhogonal to the axes of the magnet with the help of a tilt mirror, providing better MR coil integration. They further used directly an optical fiber inside the magnet for the return light path, which may reduce the artefacts from the relay. The resolution was 1.6μm over an unknown field of view. For MR-compatibility, the optics that is inside the magnet avoids ferromagnetic materials but it is not clear whether there are relevant magnetic distortions or interactions with the gradient and RF fields as it still had a large metal content. In both cases, optical scanning microscopy is relatively slow as it rasterizes the image, removing the advantage of real-time observation.

Another approach in the literature was to use a single-sided permanent magnet MR system with a static magnetic field *B*_0_ = 0.32T below a commercial microscope for simultaneous optical imaging and MRI [24].

As these approaches always had to take some compromises in the optical or MR performance and choose special optical arrangements with limited flexibility, our aim is to develop a classical microscope with diffraction-limited sub- μm resolution and flexible top/bottom, dark/bright field illumination for simultaneous MR and optical microscopy and does not interfere with the MR procedure. Our strategy is an MR compatible microscope that is integrated into the MR environment (Bruker Biospec 94/20 small animal MRI scanner, horizontal bore, 9.4T, maximum gradient amplitude 728 mT/m, slew rate 4570 mT/m/ms), such that we do not compromise the optical quality or versatility and achieve a higher optical resolution without the need of fluorescent markers. This dedicates, however, MR-compatible design specifications to minimize the magnetic field distortions at the sample and keep the sample at the isocenter of the magnet. While we demonstrate the microscope for conventional microscopy which does not exploit all possibilities of resolution and 3D penetration in optically dense objects we create a most general platform that can potentially also be adapted to more specialized techniques that we summarize in the outlook. To maximize the flexibility, this microscope is designed to use an external linear or quadrature RF coil and also to integrate an RF micro coil. The latter requires to observe the sample along the RF-field B_1_ (on the axis of the RF-coil, orthogonal to the constant magnetic field *B*_0_).

The design challenges are the limited space, in our case the 72mm diameter of the external coil, and the electromagnetic environment. Firstly, the *B*_0_ field will fully saturate ferromagnetic materials used in inductors or electromagnetic drives and exert forces and torques on magnetic materials. Near the entrance of the magnet, the *B*_0_ gradient reaches 30T/m [25], such that steel with a saturation of 1T will feel 3,000 times its gravitational force. At the same time, the *B*_0_ field should not be distorted as a distortion would cause a shift in the resonance frequeny. For example, this would result in a geomteric distortion in the reconstructed image given by the ratio of the field distortion to the readout gradient strength, e.g., 19μm distortion at 500mT/m gradient and 1ppm field distortion. In addition, the frequency shift could also artifacts in the intensity because of de-/constructive interference when averaging the resonance signal over a voxel. Secondly, the gradients may exert forces and their switching may induce eddy currents in the components of the microscope, disrupting electronics and causing forces and heating. E.g., a 100 mT field at 1 kHz induces 1.3 V induced voltage (EMF) in a 50 mm conductor loop. For 1 mm thick copper wire (ignoring self-induction and skin effects.), this results in 4.4kW heating and (at 500mT/m) 0.7N force or (at 9.4T) 0.9N × cm torque. Finally, the maximum RF power of our external coil is 1 kW at 400MHz, which could disturb the electronics. On the other hand, the maximum power of the resonance signal from a 1mm^3^ water volume is roughly of the order of 10pW if we take into account the induced EMF (roughly 50μV) from the polarized sample at 400 MHz. Hence, even small RF noise from the camera or other electronics could increase noise or generate artifacts in the MR measurement.

## Design

**Fig 1:**
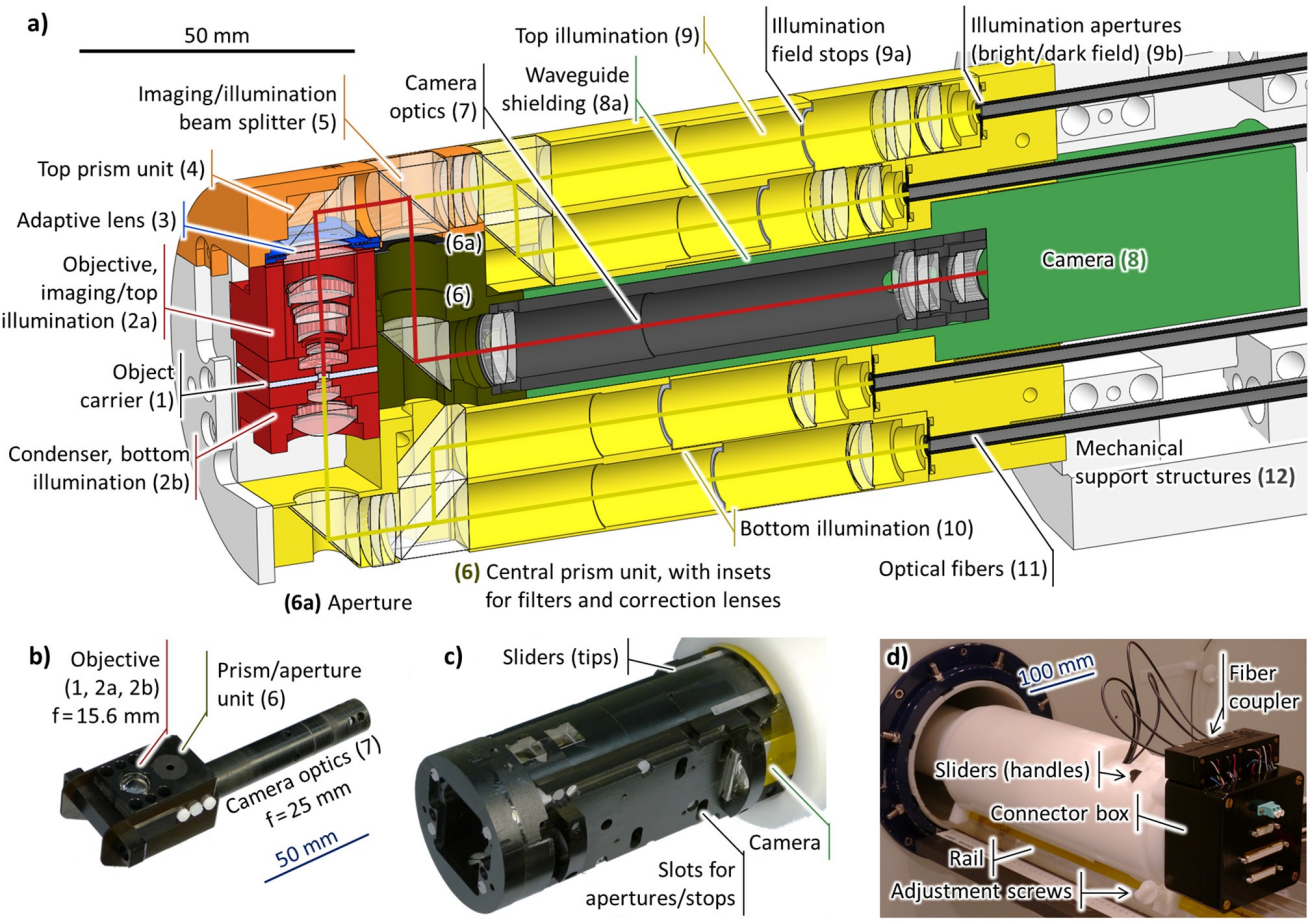
a) Color-coded cross section of the final design of the microscope. Not all lenses and apertures are to scale. The red line indicates the imaging light path and the yellow lines indicate the different illumination light paths. b) Photo of the assembled objective/prism unit/camera optics, consisting of parts (1), (2a), (2b), (6) and (7). c) Front part of the microscope. d) Rear part of the microscope when placed inside the scanner.

The overall design philosophy was to avoid moving parts, using an adaptive lens for focusing along the optical axis and fixed apertures with beam splitters to switch between different illumination modes. We obtain a large part of the design shown in Fig 1a) from general geometric considerations:

On the one hand, we chose a vertical direction of observation to be able to integrate a micro coil in the object carrier (1). On the other hand, the best optical resolution is obtained with an objective close to the sample and ideally an immersion setup—and the best MR image is obtained with the sample (1) at the isocenter of the MR scanner. This fixes the objective to the position (2) in the figure. As there will be no space for the camera above the objective, we tilted the optical axis. To allow for objectives with a large focal length, i.e. a large field of view, the prism (4) is placed at the outer bounds of the available space.

The sensor of the camera (8) is typically much larger than the object, such that its optics (7) needs a larger focal length than the objective, i.e., it needs to be oriented along the axis of the magnet. We added a further two prisms to be able to place the camera is the central axis of the magnet where it causes less artefacts.

We implemented this design with a layout consisting of a modular assembly of the imaging optics (2,7) and the sample carrier (1) and a structure containing the adaptive lens (3), illumination optics (9,10), structural support (12) and the camera (8). We milled most parts from POM (polyoxymethylene) for best machinability and structural stability, with the exception of the objective and condenser that are made from PEEK (polyether ether ketone) as it is harder and has a lower thermal expansion.

The modular imaging optics assembly (Fig 1b)) is inserted into the microscope from the front (Fig 1c)) by the user and consists of the camera optics (7) at one side and the objective (2a) above and condenser (2b) below the object (1) at the other side. They are held together by the central prism unit (6) that also carries the imaging aperture and optional optical filters or correction lenses. The 4 illumination channels are, in principle, individually configurable for dark field and bright field with apertures that are inserted from the side into the main illumination block.

The microscope is carried by a POM tube that rests outside of the scanner on a support rail that normally carries the animal beds. It may be adjusted with the help of two adjustment screws and 4 sliders that mechanically sense the position relative to the external RF coil in case it is used. At the outer end (Fig 1d)), we also find the illumination fiber coupler (see Illumination channel subsection) and a connector box.

### Optical design

We aimed to observe sample volumes between 1mm and 2mm with the highest achievable resolution and provide RGBW (red, green, blue, white) illumination in bright and dark field, top and bottom. Because of the better efficiency and higher resolution, we chose a grayscale camera sensor, with color imaging provided by reconstruction from RGB illumination. For flexibility in the imaging methods, all apertures should be configurable, the microscope should have a modular design for different magnifications and it should be possible to insert filters and correction elements.

The optical design was then constrained by four main parameters:

- To build the prototype at realistic cost, we aimed to use only lenses available from stock (plus the adaptve lens).
- Available non-magnetic adaptive lenses [26–28] provide a free aperture of 12 mm and a usable aperture around 8 to 9 mm at a focal power around ±10m^−1^. An earlier version [28] of the adaptive lens had to be oriented horizontally to avoid distortions due to gravity.
- Three dimensional MR micro-coils have aspect ratios around 0.5 [29], 1 [30] or higher [31, 32], a ratio near 1 is also desirable for passive sample containers. If a large fraction of the volume should be observed without distortions, this limits the geometric f-number to around 1.
- Taking into account tolerances for misalignments, the space above the sample is 35mm. If the tilted lightpath uses 1/2” elements, the remaining space for the objective including half of the sample volume and the adaptive lens is 22.5mm.

Further constraints came from the observing camera (see Camera and electronics Section:

- Because of the multiply tilt optical path, the optical distance from the objective to the receiving optics is ≳ 40 mm.
- The camera is shielded with a waveguide (conductive tube) with an aspect ratio of at least 4:1 (length:diameter).
- The selected camera has a resolution of 1292 × 964 with a pixel size of 3.75μm.

#### Observation channel

**Fig 2:**
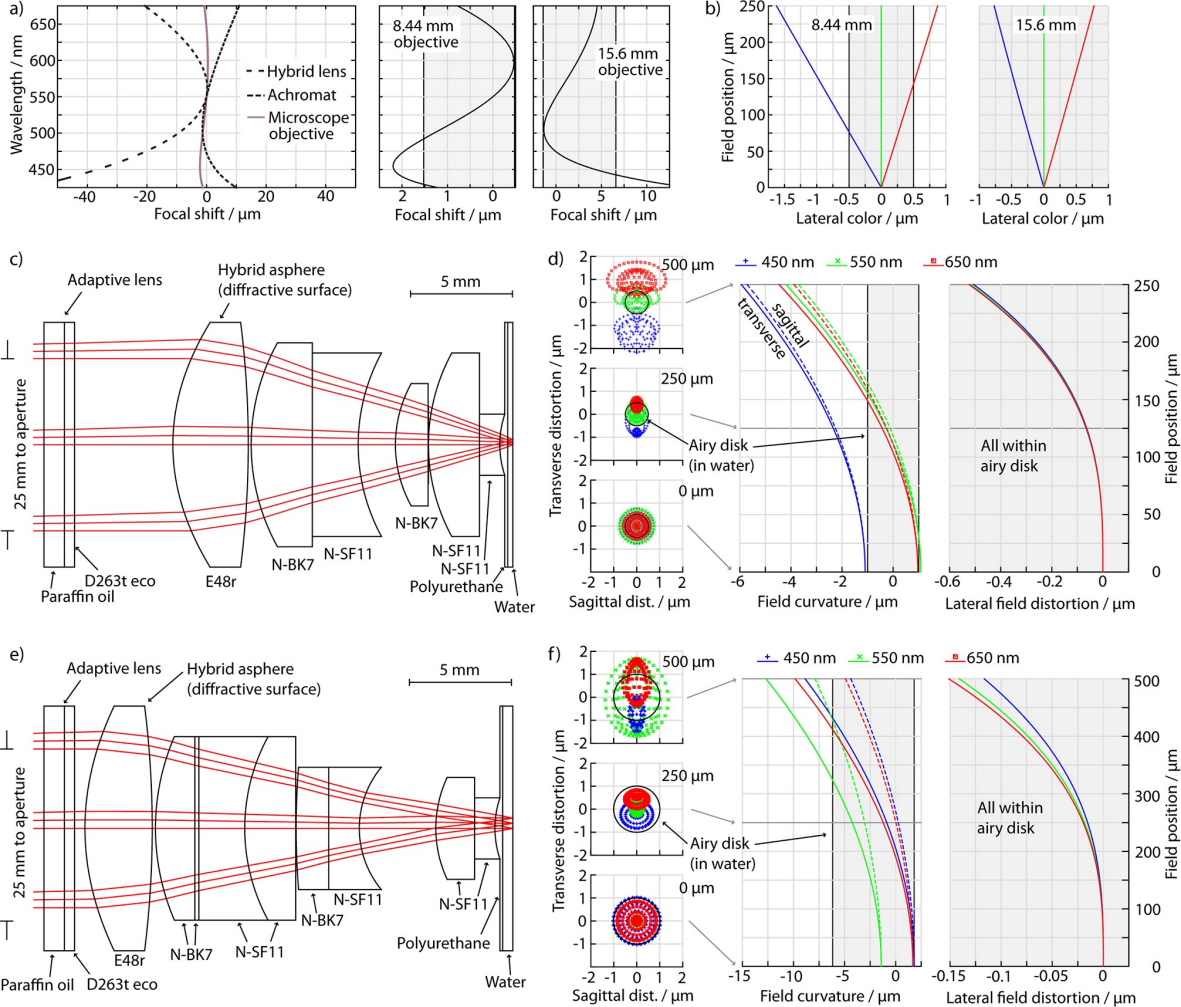
Optical simulations. Top row: a) Chromatic focal shift of a 9mm diffractive and dispersive achromat and the 8.44mm objective, focal shift of the 8.44 and 15.6mm objectives. (b) Lateral chromatic distortion for three wavelengths. Center row: c) layout and d) ray tracing of the 8.44mm objective, spot diagrams at three field positions, field curvature and field (image) distortion. Bottom: 15.6mm objective. The shaded areas indicate the Airy disk or equivalent axial positions. All simulations are reverse, so the image in the simulation corresponds to the perceived image.

A suitable geometric aperture was 8.5 mm, which still fits with the geometric requirements and allows for a large field of view. The shortest targeted focal length was hence 8.5 mm, with an option of 17 mm for a larger field of view. Since the samples are usually immersed in water or saline solutions and are not moved during the imaging, we used immersion, giving us a numerical aperture of up to NA ≃ 0.6. To achieve the corresponding resolution at least with a relatively broad-band color-LED illumination, it turned out to be necessary to go beyond a conventional achromatic design which removes the linear component of the chromatic confocal shift and implement an apochromatic color correction, canceling also the quadratic component of the chromatic focal shift. Using only commercially available stock lenses, this was realized by combining an achromatic polymer hybrid asphere with diffractive color correction and a usual achromatic combination of crown and dense flint lenses as both mechanisms have opposite signs of the remaining quadratic component of the focal shift, illustrated in Fig 2a). The optimization strategy was then as follows:

1. Choose a suitable hybrid asphere, trying out different focal lengths of the order of or longer than the desired focal power of the objective
2. Add a generic set of alternating crown and flint “lenses” (starting with flat surfaces) between the asphere and the object
3. Optimize the system under the given constraints (FoV, maximum geometric length, aperture, focal power, range of wavelengths)
4. Successively replace the singlets with similar commercially available lenses, keeping the positions of the lenses flexible
5. After replacing the last element, relax the constraint for the focal power and optimize the system with fixed NA instead

For the front lenses, we chose not the usual flint-crown combination but only dense flint, filled on the object-side with polyurethane for magnetic reasons (see Magnetic and mechanical design). The final focal lengths of the objectives after the last optimization step were 8.44 mm and 15.6 mm, respectively. The deviation of the 15.6 mm vs. 17 mm was due to the small available space of 21 mm which is already close to the focal length. The objective layouts and the resulting spot diagrams are shown in Fig 2c) to 2f). We find that the 8.44mm objective (c) is nearly diffraction-limited with an RMS spot radius (d) of 0.52μm vs. a 0.50μm Airy radius, with the exception of the outer region of the field of view where the lateral color (b) causes a small chromatic distortion for full spectrum white illumination (but not with typical color LEDs); the 15.6mm objective (e) is diffraction limited at 1.0μm throughout the field (f).

**Fig 3:**
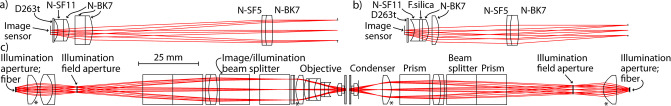
Top: 50mm (a) and 25mm (b) camera objectives. Bottom (c): Top (left) and bottom (right) bright field illumination channels. The dark field channels differ only by the absence of one prism and a different aperture. The asterisk indicates a hybrid-aspherical surface.

The camera objective was first designed for a focal length of 50mm. This gives a nominal camera resolution of 0.63μm—slightly above the optical resolution—and 0.61mm field of view for the 8.44mm objective and a 1.17μm resolution and 1.13mm field of view for the 15.6mm objective. We followed the same optimization strategy as for the microscope objectives, with the difference that we do not perform an apochromatic diffractive/dispersive color correction as shown in the layout in Fig 3a) as this is not necessary to obtain a diffraction limited image on the sensor for these longer focal lengths. To further double the field of view to 2.26mm, we also developed a camera objective with 25mm focal length that is operated with a 4.2 mm aperture (Fig 3b)). As the smaller aperture widens the limit but reduces geometric aberrations, the same objective is still diffraction limited within the larger field of view and and saturates the camera resolution. Now, the field of view is limited in the corners by the aperture of the optical elements.

#### Illumination channel

For the bottom illumination, we implemented simple condenser optics below the object with 71% of the focal length of the objectives (6.0mm and 11.1mm) but an approximately symmetric inner part because of the magnetic design. Rather than switching between light sources mechanically, the top illumination and the dark- and bright field illuminations are coupled using beam splitters as shown in Fig 1a). On the one hand, this may increase the diffuse light background in particular through coupling of the top and bottom illumination channels and hence compromise the contrast and black level in particular in dark field illumination. On the other hand, this is an MR-safe and straightforwardly realizable method.

The light path of the illumination system is similar to a Köhler illumination as shown in Fig 3c). Essentially, the illumination aperture is magnified by a factor of 6 (top) or 5.5 (bottom) and the light is then focused on the object with the objective or condenser. We use a printed photo mask with a clear disk (bright field) or annulus (dark field) as an illumination aperture. To reduce scattered light but also avoid artifacts in the image, we cut the light field in an image plane with a physically open field diaphragm. As we did not have much space in the aperture of the system to separate the dark field illumination from the imaging aperture, we used an achromatic design to ensure a consistent illumination aperture for all wavelengths.

The light is coupled into the microscope with 2 mm multimode polymer fibers. This gives a large flexibility regarding the light sources and avoids issues of their MR compatibility. It further provides a very homogeneous and isotropic light source. For the external light source, we built a fiber coupler to use RGBW LEDs (CREE XM-L) or other LEDs on star-shaped PCBs. In contrast to usual LED illumination, we do not use a PWM (pulse width modulation) brightness control, but a continuous current control to avoid image artifacts when using short exposure times or a rolling shutter.

### Magnetic and mechanical design

Biological samples are typically water-based and suspended in a water-based solution and they are smaller than the largest field of view of 2mm. While it is straightforward to correct the total value of the magnetic field in the sample region, it is not possible to correct a rapidly varying magnetic field distortion of 0.1ppm or higher to 2^nd^ order on that scale because of the limitations of the MR shimming coils [33]. Hence, the aim of the magnetic design of the objective was to minimize magnetic distortions in the sample region, assuming that it is filled with the magnetic susceptibility of water. The latter needs to be ensured in the design of the sample carriers or micro coils. A straightforward example are sample carriers made from PMMA (poly(methyl methacrylate)) with a magnetic susceptibility of χ − *χ*_*H*_2_*O*_ = −0.02ppm [34].

The main problem for the magnetic homogeneity is the strongly varying magnetic field of different optical glass types [34], in particular since the lenses have to be placed close to the sample. For this reason, chose lead-free dense flint glass (SCHOTT N-SF11, *χ* − *χ*_*H*_2_*O*_ = 0.34ppm) for the lens group closest to the sample as it comes closest to the susceptibility of water and of typical polymers; an optimal choice for the combination with polymers would have been N-SF10, which was however not available for the chosen lenses. Gaps left by the lenses were filled with clear polyurethane (−0.24ppm [34]) and the front side of the objective is protected by a 50μm thin glass (SCHOTT D263t) which should not cause distortions because of the small thickness and large aspect ratio. As a structural material, we chose PEEK (-0.30 ppm [34]) which is close in susceptibility to the polyurethane filling, has a high stiffness, relatively low thermal expansion in comparison to other un-filled polymers, good mechanical machinability and good chemical and water resistance properties. Glass reinforced polymers may have strongly varying magnetic susceptibility [34] and carbon-filled polymers may be conductive and thus affect the RF field.

**Fig 4:**
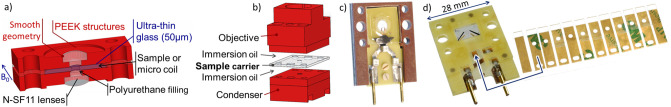
a) details of the central parts of the 8.44mm objective, b) objective assembly with PMMA sample carrier, c) adapter for a Bruker micro coil, d) Helmholtz micro coil with inserts.

The strongest discontinuity in susceptibility, however, is the one between air and material. Hence, we tried to move material edges as far away from the sample as possible and designed the innermost lens group as symmetric around the sample as optically possible, shown in Fig 4a), left. This also removes the linear variation of the magnetic field distortion over the sample, leaving only second and higher order variations.

### MR-microprobe integration

We included three different methods to acquire the MR image. The first approach is to use a simple PMMA container of different sizes as shown in Fig 4b), sandwiched in the center of the objective to carry the sample. Here, the sample is in direct water immersion contact with the objective. The image is then acquired with the external volume coil. This method promises the least magnetic distortion in the sample region and the greatest flexibility in the sample geometry at the expense of MR SNR (signal to noise ratio).

The second method is a modified Bruker planar microcoil of which we had cut the PCB to fit into the objective. To mount it without mechanical pressure, we have designed an adapter frame (Fig 4c)) that carries the vertical force from the objective and acts as a spacer because of the capacitors on the PCB. For straightforward rapid prototyping, we used a construction made from stacked layers of laser-structured FR-2 resin bonded paper sheets, joined with cyanoacrylate glue. The coil PCB is screwed into this frame and aligned with a round glass window that fills the space below the coil and fits into the cut-out window in the PCB. On top, the coil is covered with a glass window and all glass-glass interfaces are filled with halocarbon oil as a compromise to reduce optical and magnetic mismatch but avoid emitting an MR signal. This gives a better SNR and allows for good integration into the MR scanner. The coil has an inner diameter of 1.0mm and the sample volume has a diameter of 5mm and height 0.57mm, with stacks of overall 1.5mm glass below and 1.0mm above.

Finally, we also integrated the Helmholtz micro-coil chip of ref. [29] as shown in Fig 4d). For this purpose, we milled FR-4 PCBs that carry the resonance circuit and stack up to form a support frame in which the sensor was sandwiched between two laser-structured 170μm thick microscopy slides. All parts were bonded with transparent polyurethane. The sample is now carried in disposable sample carriers that are inserted into the slot between the coils from the front of the microscope without disassembly. The Helmholtz coils have 1.18mm inner diameter with a 600μm high free slot, such that we used 500μm thick PMMA foil and 300μm deep sample cavities with 0.6mm to 1.6mm diameter to either center the sample or move possible edge effects like trapped air bubbles away from the field of view. The most reliable fabrication turned out to be milling the base of the carrier and then laminating and laser-structuring (in place) 3M 9471 adhesive transfer tape and 23μm thick cellulose hydrate cover foil. The carriers are then held for filling in a custom vacuum chuck under a microscope and closed using the cover foil on the adhesive. To create optical contact and reduce magnetic susceptibility mismatch, we filled all gaps with hydrogen-free perfluoropolyether oil that we estimate to *χ* − *χ*_*H*_2_0_ ∼ 0.6ppm (extrapolated from Zeis Immersol 2010, other fluids and PTFE (polytetrafluoroethylene)) [34], that further has a refractive index close to water and that we had available in a convenient viscosity.

### Camera and electronics integration

The main requirements for the camera were a safe operation in the presence of the static *B*_0_ field and the dynamic gradient fields. Hence, it should not contain magnetic materials and also have as little metal content as possible to avoid eddy currents and vibrations. It further has to be shielded against the RF field.

We chose the Allied Vision Technologies Stingray 125B that uses a Sony ICX 445 CCD sensor [35]. It provides reliable communication in the MR environment and complete electric decoupling from the controlling computer through an optical IEEE 1394b interface. The main problem for safe operation in the *B*_0_ field are the ferrite core inductors that are subject to magnetic forces and also may not operate properly due to saturation in strong magnetic fields. They are typically found on the on-board power supply. To avoid this problem, we obtained a modified camera from the manufacturer without ferrite core inductors that has however the on-board power supply removed. Instead, we supplied all 5 required internal voltages from a custom external power supply through a cable (Sommer Cable Octave Tube) that is shielded 100% with twisted copper and aluminum-metallized fleece. To reduce the metal content, we replaced the case with a custom case made from PVC that we wrapped in 0.2mm copper foil and further sealed with bismuth-based low-temperature solder.

As the skin depth of the RF field in copper is 3.3μm, it is not possible to shield the camera in the lightpath with a transparent coating. A grid would either create shadowing effects (near the sensor) or diffraction (in the far field). Hence, we chose waveguide shielding, where the field is shielded with a long conductive tube (using the same copper foil on PVC) in which it decays exponentially on the length scale of the diameter. For a circular tube with diameter *D* much smaller than the wavelength, the electric field strength is proportional to *e*^−2*zj*_0,1_/*D*^ along the axis of the tube, where *j*_0,1_ ∼ 2.40 is the first root of the 0^th^ Bessel function of the first kind. With an aspect ratio of 4 (75mm length, 18.8mm diameter), we thus expect an attenuation of 3.8 × 10^−9^.

To avoid image artefacts and create a reproducible illumination also at short exposures, we used Recom RCD-24-1.0 constant current LED drivers that supply a controlled current up to 1*A*. The two voltage signals to control the lens are supplied with PI (Physikeinstrumente) E-836.03 OEM amplifier modules with −30 to 130V output signal and an amplification factor 10. These modules are controlled via a Measurement Computing USB-3105 16 bit, 16 channel DAC (digital/analog converter) for the illumination and a USB-3112 16 bit, 8 channel DAC for the adaptive lens from a single graphical interface written in Matlab. This software requires only minor instructions to the user and also includes the configuration of the camera and the acquisition of images and videos. We used a virtual ground to create the -3 to 13V input signal of the piezo amplifier from the -10 to 10V output of the DAC. In addition, we included a USB-2408 24 bit ADC (analog/digital converter) to potentially provide closed-loop control of the adaptive lens. For user-friendly operation, all the modules are integrated in a single housing and they are then connected through the passive filter plate into the shielded cabin of the MR scanner.

## Results and discussion

We built up the microscope as described in the design section including the 25mm and 50mm camera optics and the 15.6mm microscope objective, mostly using a 2.5 axis CNC mill and laser structuring. We also built and tested an initial prototype of the 8.44mm objective in the lab but did not adapt it to some minor geometric changes of the microscope as the larger fields of view of the 15.6mm objective were more suitable for our application.

### Optical characterization

For the optical characterization, we performed a knife edge modulation transfer function (MTF) test using a laser-structured razor blade that can be mounted at different axial and lateral positions inside a water-filled chamber in the field of view. We calibrated the magnification of the microscope at different focal positions by structuring a horizontal scale into a knife. The images were then analyzed by (1) splitting up the image into lines approximately orthogonal to the edge, (2) choosing a region of *N* = 129 pixels around a first fit of the position of the edge, (3) removing a possible linear drift of the background intensity, (4) normalizing the intensity and finally (5) differentiating the result to obtain the line spread function:

$$\begin{array}{ccc} LSF_{n} & = & \frac{I_{n+1}^{\left(norm\right)}-I_{n-1}^{\left(norm\right)}}{2\Delta x},\\ I_{n}^{\left(norm\right)} & = & \frac{I_{n}^{\left(flat\right)}+\left({\langle I_{m}^{\left(flat\right)}\rangle }_{m<N/6}-{\langle I_{m}^{\left(flat\right)}\rangle }_{m>5N/6}\right)/2}{{\langle {\left(I_{n}^{\left(flat\right)}+\left({\langle I_{m}^{\left(flat\right)}\rangle }_{m<N/6}-{\langle I_{m}^{\left(flat\right)}\rangle }_{m>5N/6}\right)/2\right)}^{2}\rangle }^{1/2}},\\ I_{n}^{\left(flat\right)} & = & I_{n}-bn, \end{array}$$

where the linear drift *b* is obtained from a first fit by $a+b\;n+f\;\text{Erf}(\frac{x-c}{d})$. Assuming that the unevenness of the edge is much smaller than the resolution, this gives us the line spread function (LSF) for every point along the edge. To obtain the RMS line width *d*, we then fitted a Gaussian curve $a+be^{-\frac{{\left(x-c\right)}^{2}}{2\;d^{2}}}$ to each line along the edge. Surprisingly, it turns out that this Gaussian fit is more robust than fitting an error function to the intensity along the line. To obtain the MTF, we performed a discrete Fourier transform of the LSF. As expected, the results of either directly transforming the LSF or transforming the normalized intensity and differentiating by Fourier transform are identical.

We measured the 15.6mm objective in the final setup at three different axial positions of the target, focused by the adaptive lens. For the 8.44mm objective, we have only preliminary data from a laboratory setup, where we focused with a set of two (concave and convex) lenses instead of the adaptive lens and measured only at one axial position. In each case, we took three images with slightly shifted foci for each point and chose the best focused one. We then dropped the highest and lowest 5^th^ percentile of the values to remove image artifacts and mis-fittings. As a small unavoidable tilt of the edge may move it partially out of focus, we chose the smallest value of a moving median over 20% of the central region of the FoV as the line width in the “inner field”, and we chose smaller value of the medians over the two outer halves of the FoV for the “outer field”. To compare the measurement with simulations, we manipulated an actual image of the knife edge to black and white with partial pixel effects along the edge and performed a geometric image simulation that we processed identically to the measurement. Finally, we obtained the diffraction broadening from a diffraction simulation and added it in quadrature to the geometric result.

**Fig 5:**
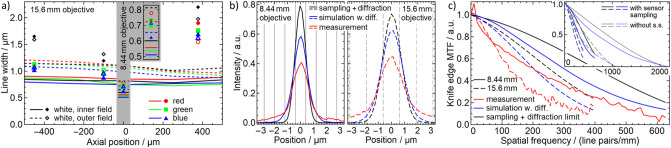
a) measured and simulated RMS line width. The data in the gray region relates to the 8.44mm objective. b) mean LSF, oversampled by a factor of 5. The grid lines indicate the pixel size. c) MTF in the central FoV and a central axial position. The inset compares the MTF from actual simulated and sampled edge images to the simulated ideal MTF.

In Fig 5a) we see that the measurements are typically 10% to 20% broader than the simulations, which may be explained by imperfect (manual) focusing, image noise, imperfections in the lens surfaces and in the fabrication and by stray light and contrast loss. In particular, the hybrid asphere has only a limited diffraction efficiency, which may result in contrast loss and may explain the broadening under white illumination. The smaller difference between inner and outer field in the measurement may result from small misalignments, which may put one of the outer regions closer to the optical axis. Similarly, the increased width at the positive axial position likely results from a small defocus of the objective, which required to focus it further than the designed focal range at this point.

To obtain a meaningful average of the LSF or the MTF, we further had to account for the tilt of the LSF along the edge. For mean of the LSF, we aligned the individual LSFs by shifting the table to the nearest pixel, and then oversampled the mean by dividing the LSFs into 5 sets according to their exact edge position. For the case of the MTF, we included a complex factor that accounts for the position *d*_*n*_ of the edge,

$$\begin{array}{c} MTF^{\left(mean\right)}\left(k\right)\;=\langle MTE_{n}\left(k\right)\;e^{-2\pi i\;d_{n}\left(k-1\right)/\left(N-1\right)}\rangle \;. \end{array}$$

In addition to the simulation we now also processed the ideal edge image and convoluted (LSF) or multiplied (MTF) it with the simulated diffraction to obtain the limitation due to sampling and diffraction. Both in the LSF (Fig 5b)) and in the MTF (Fig 5c)) we see that the simulation is very close to the sampling limit and the actual resolution of the measurement (average in inner FoV) is close to the simulation. The main difference is actually a contrast loss that we see in the outer part of the LSF and at relatively small wavenumbers in the MTF. Furthermore, we see that the resolution is essentially limited by the sampling resolution of the camera, which is not surprising as we specifically designed the objective to saturate this resolution. The slightly larger pixel size than designed of the 8.44mm objective results from a telescope effect of the focusing mechanism.

### MR and magnetic characterization

**Fig 6:**
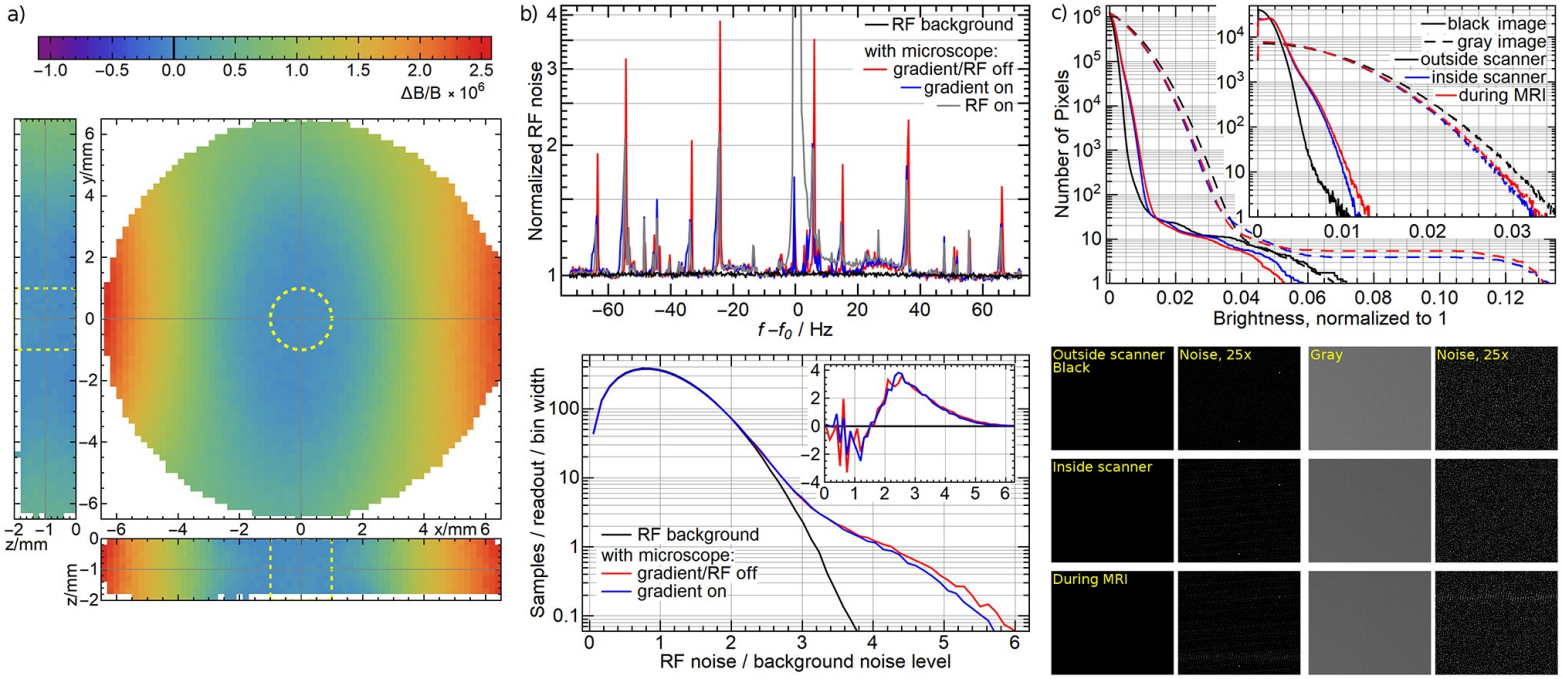
a) Distortion of the magnetic field in a 2mm thick 13mm diameter agarose phantom: Planes through the center of the maximum sample volume (dashed yellow lines), normalized to 0 at the center. Data points without signal inside the sample volume are extrapolated. Worst-case using a RF volume coil enclosing the microscope: b) Magnitude of the MR noise normalized to the average of the background noise (top) without the microscope and with the microscope, without RF and gradient fields, with the gradients turned on and with RF excitation in the presence of a small agarose phantom. Histogram of the magnitude of the MR noise (bottom), normalized such that the integral corresponds to the samples per readout; inset: difference to the background noise. c) Integrated histogram of the optical noise (main graph), histogram of the optical noise (inset) of a black image and a gray image outside of the MR scanner, inside the MR scanner (in the *B*_0_ field) and during the MR measurement. Optical sample images (segments of 143 × 121 pixels) and the magnitude of their optical noise scaled by 25.

To measure the magnetic field distortion in the sample region, we produced a water phantom with 13mm diameter cut out of 2mm thick PMMA, covered with 125μm thick polyimide film (Kapton) and filled with de-ionized water. We obtained the field map with a resolution of (0.2mm)^3^ using a a dual echo gradient echo sequence (echo times TE_1_/ TE_2_ = 2.8/ 6.8ms, repetition time TR 20 ms, flip angle 7 deg). To analyze the data, we selected the 2mm diameter, 2mm thick maximum vield of view that is marked in Fig 6a) and fitted a 4^th^ order 3-dimensional polynomial field to extract the physical field variation from the data and remove the noise. We found 59ppb peak-to-peak or 19ppb RMS field inhomogeneity, which is just slightly more than the 13ppb noise of the measurement. The result is relatively stable, with 87ppb and 17ppb when going to a 2^nd^ order polynomial or 67ppb and 20ppb at 6^th^ order. We tested the 8.44mm objective only single-sided, placing the objective without the condenser (upper part) on top of an agarose gel phantom. Despite the asymmetry, we found a similar result of 64ppb peak-to-peak or 19ppb RMS.

To determine the active effect of the microscope on the MR imaging, we measured the RF noise emitted by the camera. We chose a linear volume coil that encloses the front part microscope and is hence more sensitive to the RF noise of the microscope than the micro coil to represent the worst case. Representing a highly resolved MR measurement, we chose a bandwidth of 150kHz with 512 samples per readout. We isolated different effects by looking at the pure RF spectrum, the spectrum in the presence of an RF excitation (spin echo sequence with TE = 7ms and TR = 50ms) of a small agarose phantom (2mm by 5mm diameter) and gradient field pulses corresponding to 20mm field of view with 0.15mm resolution. In Fig 6b), we show the magnitude of the spectrum, averaged over 6272 readouts (49 repetitions × 128 planes).

We can clearly see that the baseline of the noise is not affected but there are a few discrete lines, all of which are smaller than 4× the overall noise level and they can be reduced with appropriate averaging. The lines appear to be slightly broadened and lowered in the presence of a gradient or RF field. This is probably an artifact of the averaging when there is a small shift in the noise lines due to interactions with those fields. In the presence of the RF field (gray curve), we also clearly see the hydrogen peak at *f* − *f*_0_ ≃ 0Hz from the water sample and some small spectrum near it between 10 and 20Hz, which is much smaller than the background level and may come from the structural materials. Looking at the histograms and their difference in the bottom, we see that the general noise histogram does not change but there is an added excess noise from the camera with a mean of 3× the background noise level, affecting in average approximately 5 frequency values in each individual spectrum. If not handled properly, this may increase the overall noise in the image.

We finally tested the effect of the MR environment on the optical noise and resolution of the microscope. Now, our knife edge targets made from razor blades were obviously not a suitable choice as a test target for the resolution. Hence, we structured an edge by vapor deposition of a 180μm chromium layer on glass in a semi circle with just 3mm diameter to minimize possible eddy currents in the target. This target had an intrinsic line width of 1.9μm due to the fabrication process. To test the effect of vibrations coming from the gradient field switching on the image resolution, we then acquired images with green illumination and an exposure time of 400ms while applying a 3D gradient-echo sequence (FLASH), with 30 ms repetition time, 4 ms echo time, 30° flip angle, matrix size 200 × 200 × 200, voxel size 100 × 100 × 100μm^3^, readout bandwidth 178.5 kHz. To observe the change in optical resolution resulting, the sequence was run in setup mode, i.e. a sequence interval without phase encoding (center of k-space) was repeated until manual termination. The readout gradient was played with amplitude of 413 mT/m, which is the maximum amplitude allowed by sequence implementation of the vendor. Three experiments were performed, with readout gradient in x-, y-, and z- direction, respectively. This sequence represents a realistic scenario in micro imaging and the long exposure time ensures that any vibrations will be seen as a decrease in resolution in the image. Averaging over the whole field of view, we found a loss of resolution between 0.051 and 0.085 pixels, which may be the result of small vibrations in the camera or of the lens.

To study the effect on any type of optical noise, we analyzed black images and illuminated (gray, approx. 50% saturation) images for the following 3 situations: Outside of the MR scanner, inside the MR scanner in the *B*_0_ field and during a MR measurement—again in a worst-case scenario of an RF volume coil using the same sequence. We further chose a long exposure of 100ms to capture all parts of the MR sequence. We defined the noise as the difference of a pixel brightness to the mean of its neighbors.

In Fig 6c), we show the noise histograms averaged over approx. 16 images. The main graph shows the integrated histogram, i.e., the number of pixels (y-axis) with a noise greater than a certain brightness (x-axis). The inset shows the histogram itself, i.e., the number of pixels at a certain noise level. We see that in the black image, there is a small increase in noise, whereas in the gray image the noise quantitatively decreases overall. At the far end (the ≲ 10 pixels with the highest noise), there is an increase in the gray image and a decrease in the black image. In both cases, there appears as a very faint stripe pattern as we see in the sample images in the bottom. In all cases, the dominant effect occurs already without an MR measurement, so we conclude that the *B*_0_ field has a small effect on the dynamics and the readout of the CCD sensor whereas the camera is not affected by the gradient and RF fields.

### Concurrent imaging

**Fig 7:**
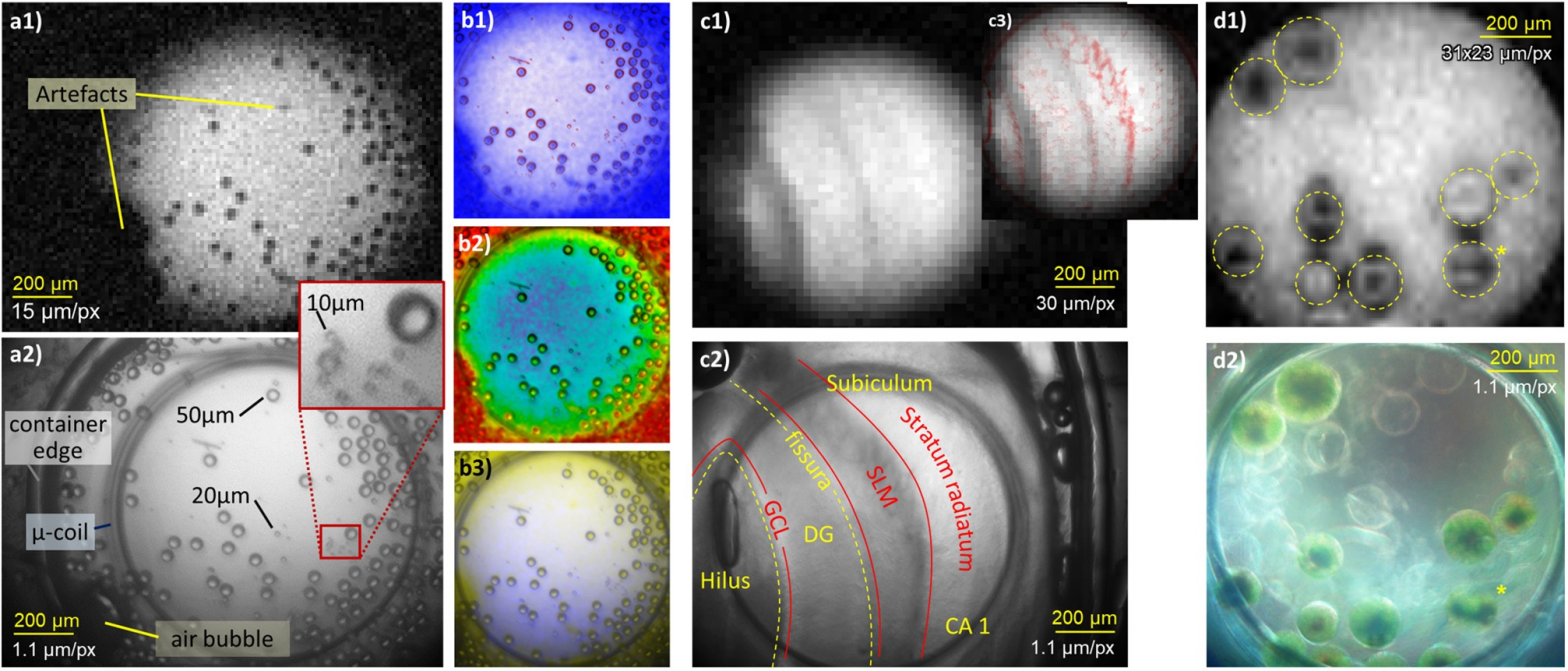
Left: MR (a1) and optical (a2) images of polymer beads suspended in water; contrast enhancement (b1), MR color coding (b2) and color addition combinations (b3). Center: MR (c1) and optical (c2) images of the hippocampus of a mouse; contrast enhancement image (c3, blue—optical, black—MR). DG—dentate gyrus, GCL—granule cell layer, SLM—stratum lacunosum-moleculare. Right: MR (d1) and optical (d2, dark field, reconctructed color) images of *eremosphaera viridis*, (*) likely formation of autospores.

We have demonstrated concurrent imaging in different situations at three examples shown in Fig 7; all MR images were acquired with the Helmholtz micro coil [29].

To illustrate the difference in the resolution, we show on the left of Fig 7 images of polymer beads of 10, 20 and 50μm suspended in water. The optical image (a2) was obtained with bottom bright field illumination and the corresponding MR image (a1) was obtained with a 2D gradient echo sequence (TR = 300ms, TE = 5.4ms, flip angle 30°) with 15μm resolution and 200μm slice thickness, FoV 2.3 × 2.5mm^2^, matrix 150 × 164, 24 averages, scan time 19:40 min. The optical image clearly resolves all beads whereas the MR image only shows the 50μm beads. We can also identify artifacts that appear in the MR image, one of which is an air bubble and the other is unknown. We further illustrate different methods to combine the images. To enhance the contrast of the MR image while still being able to differentiate between optical and MR information, we overlay the MR image (blue) with an edge detection of the optical image (red) in subtractive color combination (b1). In a color coding image (b2), we use the brightness of the optical image and code it with a rainbow map of the MR image. This is a natural representation as in nature, structures appear typically as brightness contrasts and chemical information as colors. Finally, we show an addition of complementary colors ((b3), MR: blue, optical: yellow) where the appearance of a color indicates a difference between both methods.

To illustrate just the most basic practical use of the concurrent imaging, we observed two different biological specimen. In the center of Fig 7, we show a bottom, bright field illuminated image (c2) of a slice of the hippocampus of a mouse (ex-vivo) and an MR image (c1) obtained with a TurboRARE 2D single slice sequence [36] with 30 × 30μm^2^ resolution, 300μm slice thickness, FoV 5.8 × 5.9mm^2^, matrix 192 × 197, RARE factor 8, 48 averages, TR 200 ms, TE 26 ms and scan time 3:50 min. The optical image clearly shows the major anatomical structures—the pyramidal cell layer is probably obstructed by the coil. While the MR image shows contrasts parallel to the strata, their identification is not clear. With the help of the contrast enhancement overlay image in the inset (c3), we can, however, identify the prominent dark structures as the granule cell layer and the fissura, the faint contrasts to the right of the fissura as the stratum lacunosum-moleculare and the bright region on the right as the stratum radiatum. No live animals were used in this study, the tissue sample was obtained at the Medical Center—University of Freiburg as approved by the regional council (Regierungspräsidium Freiburg) and local animal welfare officer, according to the German animal protection act.

On the right of Fig 7, we show the color reconstruction resulting from three optical images of the algae *eremosphaera viridis* with RGB bottom, dark field ilumination (d2). The background light that is apparent even after contrast enhancement probably results from the small angular difference between the illumination ring and the aperture, possible scattering at the edges of the lenses and backscattering through the beam splitters and the top illumination channels. The latter is an inherent compromise of our design concept. Yet, we can identify the internal structures of the cells at different stages of growth, and empty membranes. The chloroplasts—that are in the dark located close to the surface—appear to be contracted to different degrees due to the optical illumination. One cell (bottom right, labeled with (*)) appears to be in the formation of autospores. The MR image (d1) was obtained with a 3D balanced SSFP sequence with 2 averages, 30° flip angle and 31 × 23 × 78μm^3^ resolution FOV 8 × 6 × 5mm^3^, matrix 256 × 256 × 64, TR 13ms, TE 6.5ms, scan time 7:52 min. With the help of the optical image, we see that the chloroplasts of the algae give some MR contrast. There are further characteristic artifacts at the membranes or surrounding live cells. They appear typical for the brightness artefacts caused by magnetic dipoles in combination with the bSSFP frequency sensitivity and may result from a susceptibility mismatch of the cell plasma, possibly due to oxygen produced in photosynthesis.

In all of these cases, we see how the optical image helped to identify the structures in the MR image and interpret features in the MR image. In the glass beads, one could have misidentified noise at the resolution limit of the MR image as beads, so the optical image helped because of its resolution. For the hippocampus, it helped not only because of the resolution but also because the optical image showed structures that were not apparent in the MR image and without the optical image, we could have easily misidentified them (in fact, we had, before we rigorously registered the two images). We would not have been able to identify the structures in the MR image of the algae at all without the help of the optical image. Here, we were also helped by the color information. We further dealt with live objects. If we had taken the MR and optical images sequentially, a cell could have split or moved between optical and MR imaging, and also the structure of the cells could have changed due to different levels of illumination.

## Summary, conclusions and outlook

We have successfully demonstrated that a high quality optical imaging platform can be directly integrated into a high-field MR-scanner to perform concurrent MR and optical microscopy. In contrast to approaches in the literature, no compromises had to be made in the MR or optical imaging. Our design strategy included a multiply tilted lightpath for a compact design in combination with a transverse direction of view to be able to observe along the axis of MR micro coils or alternatively use external MR volume coils.

It provides a fully-featured conventional microscope, comparable in performance to a typical high-quality tabletop laboratory microscope. The modular optical design with each two microscope and camera objectives uses a combination of dispersive and diffractive chromatic correction to achieve a plano-apochromatic color correction with cylindrical fields of view between 0.6mm and 2.3mm and a designed resolution between 0.6μm and 2.3μm. The knife edge test in the optical characterization then showed a small broadening of 10% to 20% compared to the simulation and some loss of contrast. We designed a versatile illumination with four (top, bottom × bright, dark field) channels that can be configured with fixed apertures and are coupled into the microscope from external RGBW LEDs using optical fibers and beam splitters. The illumination, camera and focusing lens can be controlled in a software with a single GUI.

We achieved the MR compatibility with the use of polymers for structures, a modification and shielding of the camera and focusing with a piezo-driven adaptive lens. Near the object, we minimized magnetic distortions with a suitable choice of the materials with regard to their magnetic susceptibility and a symmetric and smooth design; achieving an RMS distortion of the *B*_0_ field of just 19ppb as shown in the MR and magnetic characterization. In a worst-case scenario that covers or exceeds typical MR experiments, we could produce small artifacts in both the optical and MR images using an RF-volume coil, but they are practically not relevant when imaging with micro coils or performing suitable averaging.

We designed different sample holders and adapters to perform the MR imaging either with an external animal coil or with two different (one planar and one Helmholtz) microcoils that can be mounted in the sample region of the microscope. While we already successfully used all of these MR imaging options, we showed three examples using the Helmholtz coil in detail—polymer micro beads, a hippocampus (ex-vivo) and algae cells. There, we showed different illumination modes, color reconstruction and different methods to combine the images, which allowed us to identify structures in the MR image with the help of the optical image. While this might have been possible for a fixated object also with an external microscope, the freely floating algae certainly require an in-situ optical image.

In this paper, we have demonstrated the identification of structures and artifacts in the MR image from the optical image and we performed the registration of the optical and MR images manually using the structure of the micro coils. A next development aim is to perform an automatic registration using MR and optical markers at two levels in the sample carriers. Already a clear geometric identification will be an advantage of the concurrent imaging and the optical resolution will help with the identification of structures as we saw with the mouse hippocampus. The color imaging or in the future fluorescence or spectroscopy further provides an additional channel of information. Identifying structures optically may not only help MRI but may also bring spatially resolved MR-spectroscopy [37] to a new level of utility. The second advantage is the high real-time speed of the optical imaging. Our example of live algae already shows that it helps to identify the current state of a live organism during the MR measurement, but real/time observation may also help the MR measurement. If we continuously monitor the object during a longer measurement with many averages, as it may be necessary to obtain high MR resolutions, we can perform retrospective gating [38] or sorting of the individual MR images. We can also use the concurrent imaging to perform joint contrained image reconstruction to speed up the MR measurement (or obtain a greater MR resolution in the same time) [39, 40]. Another direction would be the observation of biological or chemical processes through MRI or real-time MR-spectroscopy [41, 42] where the real-time optical imaging may help with the temporal identification.

While the optical performance is already better than other approaches to MR/optical concurrent imaging, our imaging platform can also be modified straightforwardly for different optical imaging methods that can further improve the contrast of biological specimen or provide three dimensional resolution—which improves also the contrast in three-dimensional specimen. Fluorescence microscopy with excitation in the visual range or phase contrast microscopy can be performed without structural modifications; the use of vastly different wavelengths or illumination powers such as two-photon fluorescence microscopy might require some changes in the optical materials or in the light source which is safely located outside of the scanner. Scanning methods such as confocal microscopy or optical coherence tomography could be performed by integrating compact, non-magnetic piezo-actuated adaptive prisms [43, 44], while the adaptive lens can also be used for fast axial scanning [45] and for aberration correction [46].

## Supporting information

S1 Fig (TIFF)
