# Supplementary figures and images for: MR-compatible optical microscope for in-situ dual-mode MR-optical microscopy
Source: PLoS One. 2021 May 10;16(5):e0250903. doi: 10.1371/journal.pone.0250903 (PMC8109821; doi:10.1371/journal.pone.0250903)

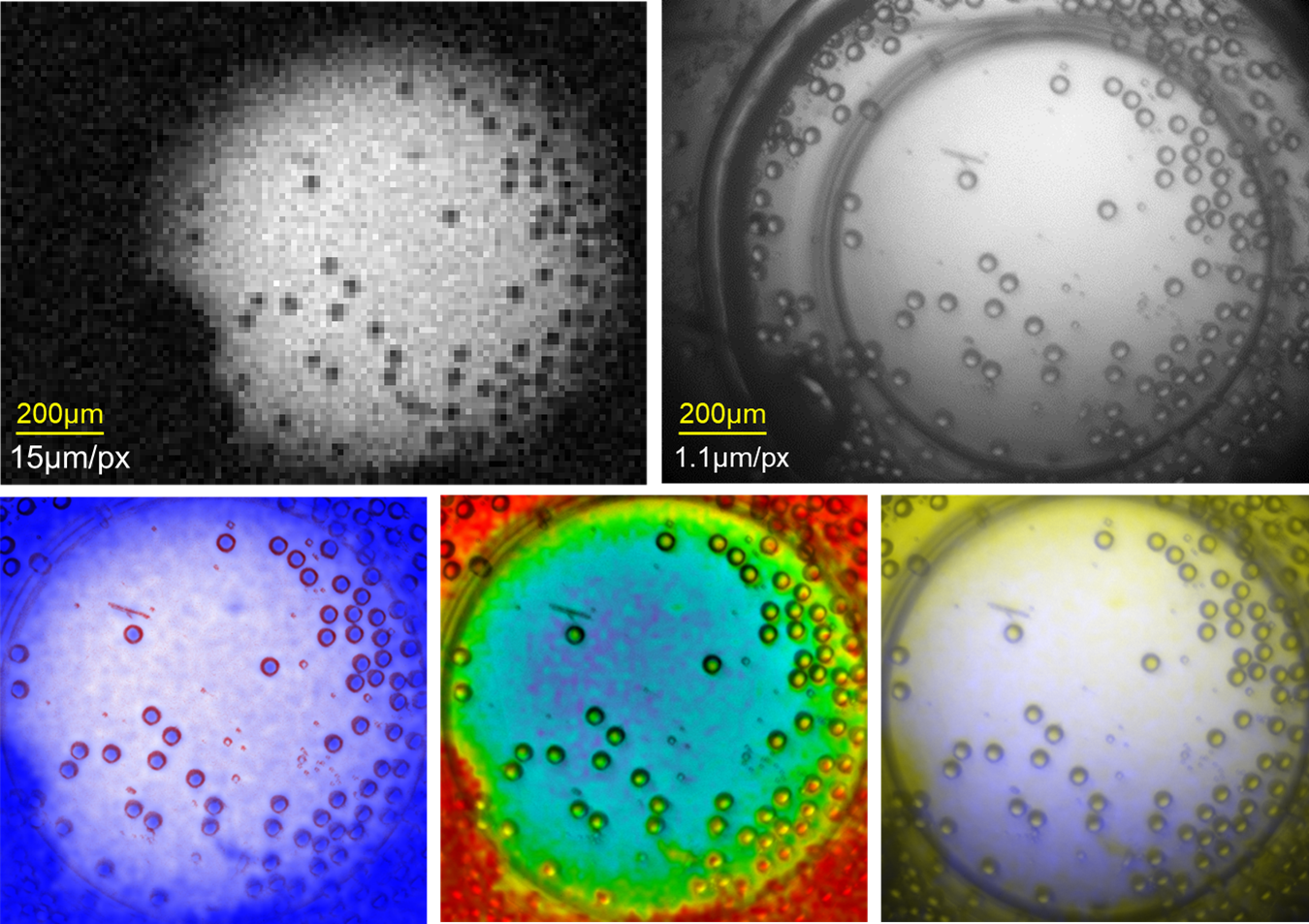

Supplement: S1 Fig — (TIFF) [file pone.0250903.s001.tiff]
